# Changes in serum adiponectin concentrations in critical illness: a preliminary investigation

**DOI:** 10.1186/cc7941

**Published:** 2009-07-02

**Authors:** Bala Venkatesh, Ingrid Hickman, Janelle Nisbet, Jeremy Cohen, John Prins

**Affiliations:** 1 Department of Intensive Care, Princess Alexandra & Wesley Hospitals, University of Queensland, Ipswich Road, QLD 4102, Woolloongabba, Australia; 2 Department of Endocrinology, Princess Alexandra Hospital, University of Queensland, Ipswich Road, QLD 4102, Woolloongabba, Australia; 3 Department of Intensive Care, Royal Brisbane Hospital, University of Queensland, Butterfield Street, Herston Road, Herston, QLD 4029, Brisbane, Australia

## Abstract

**Introduction:**

Adiponectin plays an important role in the regulation of tissue inflammation and insulin sensitivity. Perturbations in adiponectin concentration have been associated with obesity and the metabolic syndrome. Data on adiponectin pathophysiology in critical illness are limited.

**Methods:**

Twenty three critically ill patients (9 severe sepsis, 7 burns, 7 trauma). Adiponectin assays on Days 3 (D3) and 7 (D7). Simultaneous, cortisol, cortisone and CRP measurements. Data from 16 historical controls were used for comparison.

**Results:**

The mean plasma adiponectin concentration for the ICU cohort on D3 and D7 were not significantly different (4.1 ± 1.8 and 5.0 ± 3.3 mcg/ml respectively, *P *= 0.38). However, these were significantly lower than the mean plasma adiponectin in the control population (8.78 ± 3.81 mcg/ml) at D3 (*P *< 0.0001) and D7 (*P *= 0.002). Plasma adiponectin showed a strong correlation with plasma cortisol in the ICU group on both D3 (R^2 ^= 0.32, *P *< 0.01) and D7 (R^2 ^= 0.64, 0.001). There was an inverse correlation between plasma adiponectin and CRP on D7, R = -0.35.

**Conclusions:**

In this preliminary study, critical illness was associated with lower adiponectin concentrations as compared with controls. A significant relationship between plasma cortisol and adiponectin in critically ill patients was evident, both during the early and late phases. These data raise the possibility that adiponectin may play a part in the inflammatory response in patients with severe illness.

## Introduction

Adiponectin, a hormone secreted exclusively by adipose tissue, plays an important role in the regulation of tissue inflammation and insulin sensitivity [1]. Perturbations in circulating adiponectin concentrations are associated with the metabolic syndrome, altered inflammatory response and insulin resistance [2]. Hypoadiponectaemia is also associated with impaired endothelium-dependent vasorelaxation [3]. Although several of the above features are also evident in human critical illness, the underlying mechanisms are not fully understood. Some of these manifestations have been attributed to changes in plasma cortisol profile.

Data in patients with viral infections and human experimental endotoxaemia suggest altered release patterns of adiponectin in these states [4,5]. However, there are no published data on circulating serum adiponectin concentrations in human septic shock and critical illness. We therefore utilised available samples from a previously undertaken study of critically ill patients to examine serial changes in serum adiponectin concentration in a heterogeneous cohort of critically ill patients (sepsis, trauma and burns), determine the relation between the inflammatory response and adiponectin concentrations, and evaluate the correlation between plasma cortisol and adiponectin concentrations.

## Materials and methods

The plasma samples for this study were obtained from our previously published study investigating plasma cortisol-cortisone ratios in 52 critically ill patients comprising of three cohorts – burns, trauma and sepsis [6]. Residual plasma samples for adiponectin analysis were only available in 23 of these patients (nine sepsis, seven trauma, seven burns; age range 26 to 65 years; 21 males and 2 females), which were used in the present study. An independent ethics committee approval was obtained from the Royal Brisbane Hospital Ethics Committee for this study and reporting of data. The original samples were collected after informed consent from either patients or their next of kin. The other measurements on the same samples from these patients performed in the original study (cortisol, cortisone and C-reactive protein (CRP)) were used for correlative analysis.

A detailed description of inclusion and exclusion criteria was provided in the original paper. Briefly, patients with septic shock (as defined in Consensus Criteria), burns of more than 30%, and blunt or penetrating trauma of at least two body regions requiring admission to the critical care unit were enrolled in the study.

Patients younger than 16 years of age, those with a previous history of adrenal or pituitary disease, prolonged use of oral or inhaled glucocorticoids or current therapy with any such agents were excluded. The care of the patients was as per standard practice. No patient received intravenous or oral glucocorticoids.

In the original study, blood samples were collected for analysis of cortisone, cortisol and CRP daily for the first five days and on days 7, 10, 15 and 28. Residual sera were stored in a freezer at -20°C. As the predominant number of residual samples, was available only on day 3 (D3) and D7, these samples were used for the adiponectin assay.

### Biochemical measurements

Total serum adiponectin was measured using human adiponectin radioimmunoassay (Linco Research, St Charles, MI, USA; coefficients of variation (CV) for the assay <10%). Cortisol and cortisone were measured by high performance liquid chromatography. The CV at cortisol levels of 23 nmol/l and 1006 nmol/l were 6.5% and 2.4%, respectively, and for cortisone at concentrations of 110 nmol/l and 1026 nmol/L were 10.9% and 9.2%, respectively. CRP (standard assay) was assayed using an immunoturbimetric assay (Roche Diagnostics, Sydney, Australia). Interassay CV at 13 mg/L was 4.5%.

### Statistical analysis

The means and standard deviations for normals obtained from population values from our laboratory (see Results section) were used to compare the patient populations. Continuous, normally distributed variables were summarised as mean ± standard deviation. Changes in plasma adiponectin between D3 and D7 were compared using an unpaired T-test assuming unequal variances. The degree of association between variables (adiponectin and cortisol, cortisone, CRP and cortisol/cortisone ratio) and skewed or ordinal outcome measures (such as Acute Physiology and Chronic Health Evaluation (APACHE) and simplified acute physiology score (SAPS)) was assessed using Spearman's correlation coefficient (r_s_). Statistical significance was taken at a level of 5%.

## Results

The demographic profile, the diagnostic categories and the plasma endocrine profile of the patients are presented in Table 1.

**Table 1:** Demographic, diagnostic, sickness severity and endocrine profiles of the study group

Category	APACHE	Hospital survival	D3 Adiponectin	D3 Cortisone	D3 Cortisol	D3 CRP	D7 Adiponectin	D7 Cortisone	D7 Cortisol	D7 CRP
Sepsis	10	Survived	4.7	24	238	187	4.6	33	321	146
Sepsis	16	Survived	1.6	34	331	223	3.1	33	413	85
Sepsis	22	Survived	4.4	26	462	286	7.4	24	490	301
Sepsis	8	Survived	4.4	19	97	147	2.6	31	444	403
Sepsis	19	Survived	1.0	23	288	265	2.4	31	325	204
Sepsis	28	Survived	2.4	N/A	N/A	447	3.4	13	323	266
Sepsis	24	Survived	5.1	21	173	202	8.9	23	998	94
Sepsis	14	Died	4.4	14	451	264	3.3	13	230	166
Sepsis	13	Survived	8.3	93	2620	278	14.3	35	1770	166
Trauma	12	Survived	2.9	17	371	347	3.6	12	336	184
Trauma	19	Survived	N/A	32	159	109	3.1	28	352	N/A
Trauma	15	Survived	4.7	36	162	266	N/A	2	463	76
Trauma	12	Survived	3.5	50	379	224	5.6	4	83	178
Trauma	12	Survived	6.0	62	681	N/A	7.0	14	517	76
Trauma	10	Survived	7.4	11	264	324	2.3	N/A	N/A	564
Trauma	18	Survived	4.8	42	588	258	N/A	N/A	N/A	N/A
Burns	15	Unknown	4.2	22	180	N/A	N/A	N/A	N/A	N/A
Burns	7	Survived	N/A	13	364	N/A	9.1	36	279	154
Burns	16	Survived	2.8	36	368	218	N/A	25	372	410
Burns	19	Unknown	4.0	28	135	104	3.0	17	217	198
Burns	17	Survived	3.2	26	262	202	5.3	35	411	376
Burns	25	Survived	N/A	34	311	241	2.8	17	197	251
Burns	24	Died	2.8	27	322	144	2.7	16	283	228

Cortisol and Cortisone expressed in nmol/.

Adiponectin expressed in mcg/ml.

APACHE = Acute Physiology and Chronic Health Evaluation; CRP = C-reactive protein; N/A = not available.

### Plasma adiponectin profiles

The mean plasma adiponectin concentration for the whole cohort on D3 and D7 were 4.1 ± 1.8 and 5.0 ± 3.3 mcg/ml, respectively (*P *= 0.38). The mean plasma total adiponectin in the control population (16 historical controls; 12 males, 4 females, mean age 38.9 ± 8.7 years) was 8.78 ± 3.81 mcg/ml, significantly higher than the total intensive care group at D3 (*P *< 0.0001) and D7 (*P *= 0.002).

### Plasma cortisol and cortisone profile

There were no differences in plasma cortisol (418 ± 512 vs 441 ± 362 nmol/L, *P *= 0.91) or plasma cortisone (31 ± 18 vs 22 ± 11 nmol/L, *P *= 0.06) between D3 and D7, respectively.

### Relation between plasma adiponectin vs inflammatory markers

**Figure 1:**
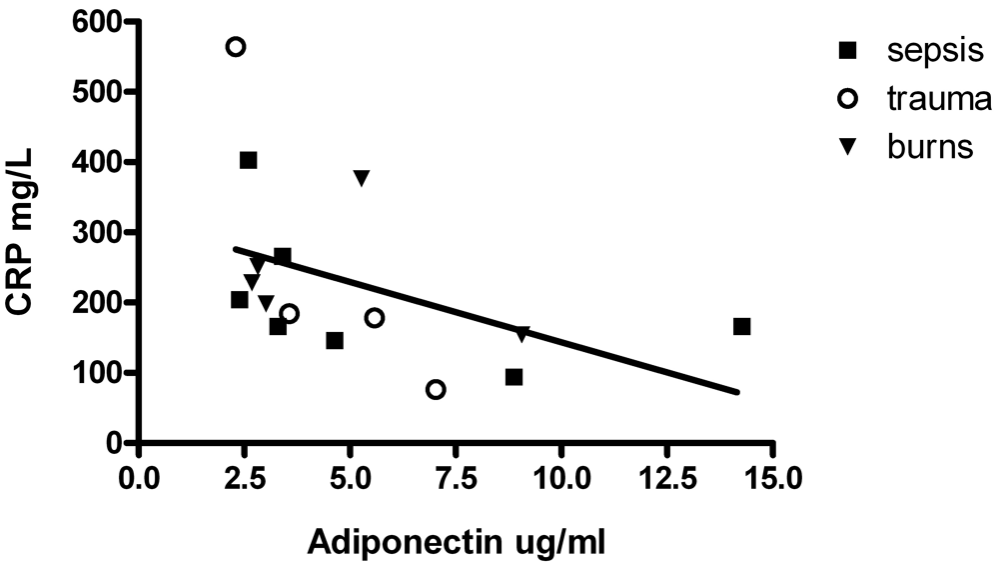
The relation between serum adiponectin and serum C-reactive protein (CRP) on day 7. A regression line is shown.

There was a poor correlation between plasma adiponectin and CRP on D3; however, on D7 an inverse correlation was noted, R = -0.35 (Figure 1).

### Relation between plasma adiponectin and sickness severity

There was a trend towards a relation between APACHE II scores and plasma adiponectin on D3 (R = 0.4, *P *= 0.07), but not on D7 (R = 0.17, *P *= 0.48).

### Relation between plasma adiponectin and cortisol and cortisone

For correlative analysis, missing results were removed and only those with matching adiponectin and adrenal hormonal data were included. Plasma adiponectin showed a strong correlation with plasma cortisol in the group as a whole on D3 (R^2 ^= 0.32, *P *= 0.01) and D7 (R^2 ^= 0.64, *P *= 0.0001).

## Discussion

To the best of our knowledge this is the first report of plasma adiponectin profiles in a heterogeneous cohort of critically ill patients. In this study, we have demonstrated a lower plasma adiponectin concentration as compared with historical controls and a strong association between plasma cortisol and adiponectin. This is notable because plasma cortisol concentrations vary widely in critically ill adults owing to the heterogeneity of the stress response. A trend towards an inverse relation between the inflammatory response and adiponectin, and a linear response between sickness severity and plasma adiponectin was also observed.

The mechanism behind the reduction in plasma adiponectin was not investigated in this study. However, it is well recognised that glucocorticoids, inflammation and oxidative stress (commonly associated with critical illness) are known to decrease adiponectin production [7]. This pattern is also consistent with what has been observed in rodent models of sepsis [8]. A significant positive relation between plasma cortisol and adiponectin has been previously shown in healthy volunteers [9,10], particularly in males [10]. In our cohort, more than 90% were males, and this strong association was evident even in critical illness in our study in both the early and late phases. A possible mechanism for the relation between cortisol and adiponectin could be that the promoter region for the adiponectin gene contains consensus sequences for glucocorticoid receptor binding [11]. The inverse association between adiponectin and CRP (R = -0.35) in our study is consistent with what has been reported in patients with coronary artery disease [12].

### Significance in critical illness

As adiponectin plays an important role in tissue inflammation, endothelial function and vascular reactivity, this could represent a key pathway in determining steroid and inotrope responsiveness in septic shock. Adiponectin, through its negative feedback effects on TNF-alpha [13], may be a critical determinant of the severity of the inflammatory response and multiple organ dysfunction. In animal models of sepsis, adiponectin modulates inflammation and survival [14]. However, its role in human critical illness needs to be evaluated further.

### Limitations

This is a preliminary study limited by a small sample size. Adiponectin analyses were performed on residual sera from our previous study [6] and historical controls were used. Comparisons between D3 and D7 are partly limited because paired samples were not available in every patient. As body mass index (BMI) is known to be associated with plasma adiponectin, a correlation between the BMI of patients and adiponectin would have provided additional useful information; however, as body weights are not routinely measured in critically ill patients, this analysis was not possible. Despite these limitations, the data from this study are in keeping with similar plasma profiles of adiponectin in human volunteers administered endotoxin [5]. Moreover, the results from D3 and D7 are relevant as patients would have completed their resuscitation phase and therefore large fluid shifts are less likely in this stage to have an impact on plasma concentrations.

## Conclusions

In conclusion, in this preliminary study, we have demonstrated a significant relation between plasma cortisol and adiponectin in critically ill patients, both during the early and late phases. Overall, these data raise the possibility that adiponectin may play a part in the inflammatory response in patients with severe illness. These results are preliminary and hypothesis generating. The relation between adiponectin and the inflammatory response, organ dysfunction and outcome in critical illness should be the subject of future investigations.

## Key messages

• Adiponectin a hormone secreted exclusively by adipose tissue has an important role in the regulation of tissue inflammation and insulin sensitivity.

• In this preliminary study of critically ill patients, serum adiponectin concentrations were reduced and were correlated with inflammatory markers such as CRP and plasma cortisol.

• Overall, these data raise the possibility that adiponectin may play a part in the inflammatory response in patients with severe illness.

## Abbreviations

APACHE: Acute Physiology and Chronic Health Evaluation; BMI: body mass index; CRP: C-reactive protein; CV: coefficient of variation; D3: day 3; D7: day 7; SAPS: simplified acute physiology score; TNF: tumour necrosis factor.

## Competing interests

The authors declare that they have no competing interests.

## Authors' contributions

BV: Study design, analysis of data and manuscript preparation. IH: Adiponectin data analysis and manuscript review. JN: Adiponectin data analysis and manuscript review. JC: Cortisol and adiponectin analysis and manuscript review. JP: Study design, analysis of data and manuscript preparation.
